# SNAP-Tag-Based Antibody–Drug Conjugates Targeting Epidermal Growth Factor Receptor 1, Epidermal Growth Factor Receptor 2, Trophoblast Cell-Surface Antigen 2, and Tissue Factor for Ovarian Cancer Treatment

**DOI:** 10.1021/acsomega.5c10377

**Published:** 2026-03-10

**Authors:** Chaoyu Zhang, Wenjie Sheng, T. M. Mohiuddin, Marwah Al-Rawe, Roland Schmitz, Marcus Niebert, Felix Zeppernick, Ivo Meinhold-Heerlein, Ahmad Fawzi Hussain

**Affiliations:** † Department of Gynecology and Obstetrics, Medical Faculty, 9175 Justus-Liebig-University Giessen, Klinikstr. 33, Giessen 35392, Germany; ‡ Medizinische Hochschule Brandenburg Campus GmbH, Hochstraße 29, Brandenburg an der Havel, Brandenburg 14770, Germany; § Institute of Pathology, University Hospital Giessen, Justus-Liebig-University Giessen, Langhanssstr. 10, Giessen 35392, Germany

## Abstract

The ovarian cancer is a heterogeneous and most malignant form of gynecologic cancer. Despite surgical intervention and systemic chemotherapy, treatment options for this cancer remain limited. Antibody–drug conjugate (ADC) represents a novel targeted therapy that uses an antibody to specifically deliver toxins to tumor sites. With the approval of the first ADC targeting ovarian cancer, more ADCs are currently under preclinical investigation or clinical trials to expand therapeutic options. In this study, we developed four ADCs targeting epidermal growth factor receptor (EGFR), epidermal growth factor receptor 2 (Her2), trophoblast cell-surface antigen 2 (Trop2), and tissue factor (TF) that are highly expressed on ovarian cancer cells. Our ADCs are constructed using single chain antibody fragments (scFvs) as the antibody backbone, with the cytotoxic agent monomethyl auristatin E conjugated via SNAP-tag technology, offering a high site-specific conjugation efficiency. All four ADCs preliminarily demonstrated specific binding and internalization, as verified by flow cytometry and fluorescence microscopy. Additionally, the ADCs exhibited potent and specific cytotoxicity in a dose-dependent manner in four ovarian cancer cell lines, inducing apoptosis at nanomolar concentrations. These constructs showed encouraging preliminary characteristics, although further studies are required to validate their therapeutic potential.

## Graphical abstract

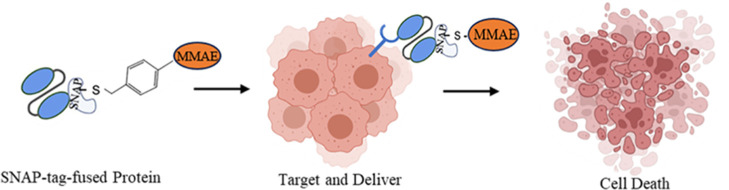

## 1 Introduction

Ovarian cancer is one of the most common cancer types in gynecologic cancers, with an estimated 313,959 new cases diagnosed in 2020.^1^ Although its incidence is lower than those of other cancers, ovarian cancer is the most lethal gynecologic malignancy. Approximately two-thirds of patients with epithelial ovarian cancer, the most common subtype and accounting for 90% of cases, are diagnosed at an advanced stage (stages III–IV).^2–5^ While cytoreductive surgery and chemotherapy remain the standard treatments of ovarian cancer, emerging therapies, such as targeted therapy, hormone therapy, and immunotherapy, are becoming increasingly important.

Targeted therapy is one of the most promising approaches to reduce the side effects caused by traditional chemotherapy as the small molecules or macromolecules precisely target the tumor cells rather than indiscriminately attacking fast-dividing cells. Olaparib, a poly (adenosine diphosphate-ribose) polymerase (PARP) inhibitor, has been widely used as maintenance therapy for ovarian cancer, either alone or in combination with bevacizumab, achieving satisfactory outcomes.^6,7^ However, the availability of targeted antibodies remains limited not only for ovarian cancer but also for other cancer types. This limitation arises because most antibodies target the antigens presented on the cell surface, but not all of these antibodies exhibit sufficient cytotoxicity to be translated to clinical applications. As a result, enhancing the toxicity of the monoclonal antibody (mAb) has been a significant challenge in targeted therapy. An alternative strategy to address this challenge is using mAb as a vehicle to deliver highly potent cytotoxic agents specifically to tumor cells rather than increasing the inherent therapeutic properties of mAbs themselves.

The antibody–drug conjugate (ADC) consists of a mAb with high specificity for tumor-associated antigens on the cell surface, a potent cytotoxic payload, and a stable linker to tether the drug to the mAb. This technology expands the clinical application of mAbs by using them as delivery vehicles rather than as therapeutic agents to transport cytotoxic agents directly to the tumor sites. As of 2024, 15 ADCs have been approved globally.^8^ However, mirvetuximab soravtansine (MIRV, Elahere), which targets folate receptor-α (FRα), remains the first and also the only ADC approved for ovarian cancer treatment. Although FRα is widely overexpressed in ovarian cancer,^9,10^ a subset of patients does not benefit from MIRV therapy. Additionally, the identification of alternative targets is crucial for providing new treatment options for patients who develop drug resistance. Despite their promise, ADCs are also facing several challenges, including poor tumor penetration, heterogeneous product composition, and the development of drug resistance.^11,12^

The limited tissue penetration of ADCs is largely due to the size of the full-length antibody, which is approximately 150 kDa. To address this limitation, smaller antibody fragments such as single-chain variable fragment (scFv) and antigen-binding fragments (Fab) are promising alternatives for developing a smaller ADC format due to their reduced size. Numerous studies have evaluated the drug delivery properties of these fragments.^13^ Conjugation methods are also evolving, with increasing focus on producing homogeneous ADCs to improve pharmacokinetic profiles. One emerging approach is SNAP-tag technology, a modified form of the human repair protein O6-alkylguanine-DNA-alkyltransferase. This enzyme-based and site-specific conjugation method takes advantage of the tag’s self-labeling ability via forming a stable thioether bond between the tag and benzylguanine (BG) derivatives.^14^ In addition to FRα, other tumor-associated antigens such as epidermal growth factor receptor (EGFR), epidermal growth factor receptor 2 (Her2), trophoblast cell-surface antigen 2 (Trop2), and tissue factor (TF) are also overexpressed in ovarian cancer. Approximately 70%, 53%, 92%, and 96% of ovarian carcinomas exhibit overexpression of EGFR, Her2, Trop-2, and TF, respectively.^15–22^ These antigens are associated with aggressive clinical features, highlighting their potential as candidate targets for ADC development.

In this study, we contrived to generate ADCs by integrating scFv and SNAP-tag technology. This approach is intended to reduce the recombinant protein size to improve tissue penetration and enable the production of homogeneous ADCs for better pharmacokinetic properties. We generated four scFv-SNAP-tag fusion proteins targeting EGFR, Her2, Trop2, and TF: 1) scFv-Erbitux-SNAP derived from anti-EGFR mAb Erbitux;^23^ 2) scFv-Herceptin-SNAP derived from anti-Her2 mAb Herceptin;^24^ 3) scFv-Sacit-SNAP derived from anti-Trop2 ADC sacituzumab govitecan;^25^ and 4) scFv-Tisot-SNAP, derived from anti-TF ADC tisotumab vedotin.^26^ The specific binding, rapid internalization, and selective cytotoxicity of these recombinant SNAP-tag-fused proteins were confirmed *in vitro* by conjugating with fluorescent dyes (SNAP-Surface Alexa Fluor 647) or monomethyl auristatin E (MMAE). The four MMAE-based ADCs showed the potential to be further validated and developed for ovarian cancer-targeting treatment.

## 2 Materials and Methods

### 2.1 Cell Culture

The ovarian cancer cell lines SKOV3 (HTB-77), OVCAR3 (HTB-161), A2780 (93112519), and the human embryonic kidney cell line HEK293T (CRL-11268) were purchased from the American Type Culture Collection. The OVCAR4 was kindly provided by Dr. Karen Bräutigam (Department of Gynecology and Obstetrics, University Hospital Schleswig-Holstein, Campus Lübeck) as a gift. All of the cells were cultured in RPMI 1640 medium (Biowest) supplemented with 10% (v/v) fetal bovine serum (FBS, Thermo Fisher Scientific) and 100 U/mL penicillin–streptomycin (Thermo Fisher Scientific). The cells were maintained at 37 °C in an incubator with 5% of CO_2_.

### 2.2 Protein Expression and Enrichment

The open reading frame of the scFv-SNAP fusion protein (Supporting Information Figure 1) DNA sequence was inserted into a pSecTag2-based pMS eukaryotic expression vector and transiently transfected into HEK293T cells using Roti Fect (Carl Roth, Karlsruhe, Germany) to produce the protein, as previously described.^14^ Cells were cultured in medium containing zeocin (0.1 mg/mL; InvivoGen, Toulouse, France) for selection. The fusion proteins were enriched from the cell culture supernatant using immobilized metal affinity chromatography (IMAC) with increasing concentrations of imidazole (10, 40, and 250 mM, respectively) using a Ni-NTA Superflow cartridge (Qiagen, Hilden, Germany) on an KTA start system (GE Healthcare Bio-Sciences AB, Uppsala, Sweden). To evaluate the enrichment efficiency and the activity of the scFv-SNAP fusion protein, all flow-through fractions were collected separately and incubated with SNAP-Surface Alexa Fluor 488 (New England Biolabs, Ipswich, MA, USA) at room temperature for 20 min in the dark. Samples were then analyzed by SDS-PAGE followed by Coomassie brilliant blue staining.

### 2.3 Generation of MMAE-Based ADCs

The benzylguanine-modified amino-PEG4-Val-Cit-PAB-MMAE (BG-MMAE) was generated as previously described.^27^ Briefly, the amino-PEG4-Val-Cit-PAB-MMAE (MMAE) (BroadPharm, San Diego, CA, USA) was incubated with BG-GLA-NHS (New England Biolabs, Ipswich, MA, USA) at a 1:2 molar ratio in 1× phosphate-buffered saline (PBS) for 4 h at room temperature. BG-MMAE was purified by high-performance liquid chromatography, and the mass was confirmed by using a Bruker MicroTOF LC mass spectrometer.

To generate the MMAE-based ADCs, the scFv-SNAP fusion protein was incubated with a 1.5-fold molar excess of BG-MMAE at room temperature for 2 h. Residual BG-MMAE was removed by 40K MWCO Zeba Spin Desalting Columns (Thermo Fisher Scientific, Rockford, IL, USA). The conjugation efficiency was confirmed by postincubation with SNAP-Surface Alexa Fluor 488 (New England Biolabs, Ipswich, MA, USA), followed by SDS-PAGE and Coomassie brilliant blue staining. Fluorescent and Coomassie-stained bands were visualized under UV light using a ChemiDoc XRS+ System (Bio-Rad, Hercules, CA, USA) and Odyssey DLx Imager (LI-COR Biosciences, Bad Homburg, Germany).

### 2.4 Generation of SNAP-Surface Alexa Fluor 647-Labeled scFv-SNAP

The scFv-SNAP fusion protein was incubated with a 2-fold molar excess of SNAP-Surface Alexa Fluor 647 at room temperature for 2 h in the dark. Residual dye was removed using the same desalting procedure as described in Section 2.3. Conjugation efficiency was confirmed by following the same method used for the MMAE-based ADCs.

### 2.5 Flow Cytometry

Cell surface expression of EGFR, Her2, Trop2, and TF was evaluated by flow cytometry. Briefly, 4 × 10^5^ cells were collected and washed twice with PBS and then stained with anti-EGFR (H11, 0.5 μg, Thermo Fisher Scientific), anti-Trop2 (MR54, 1 μg, Thermo Fisher Scientific), or anti-TF (CD142, 10 μL, Miltenyi Biotec) antibodies in 200 μL of PBS for 30 min on ice. According to the manufacturer’s instructions, cells were fixed by 4% formaldehyde solution at room temperature for 10 min, followed by permeabilization with 0.1% Triton X-100 in TBS at room temperature for 5 min followed by a blocking step (10% FBS and 1% BSA in PBS) on ice for 30 min before incubation with the anti-Her2 antibody (3B5, 0.2 μg, Thermo Fisher Scientific), as the antibody binds to the intracellular domain of Her2. After two washing steps, cells were incubated with goat antimouse IgG (H + L) Highly Cross-Adsorbed Secondary Antibody conjugated with Alexa Fluor Plus 647 (0.25 μg) for 30 min on ice to detect primary antibodies. After two washing steps, cells were resuspended in 200 μL of PBS.

The binding ability of the scFv-SNAP fusion protein was evaluated by flow cytometry. Similarly, 4 × 10^5^ cells were collected and washed with PBS twice and then stained with SNAP-Surface Alexa Fluor 647-labeled scFv-SNAP fusion protein (1 μg) in 200 μL of PBS for 30 min on ice. The labeling process involved incubating the scFv-SNAP fusion protein with SNAP-Surface Alexa Fluor 647 at a 1:2 molar ratio at room temperature in the dark for 2 h. After two washing steps, cells were resuspended in 200 μL of PBS.

All analyses were performed using CytoFLEX Flow Cytometers (Beckman Coulter, Indianapolis, IN, USA), and data were analyzed in FlowJo 10.7.1 (Becton, Dickinson & Company, Ashland, OR, USA).

### 2.6 Fluorescence Microscopy

SKOV3, OVCAR3, and A2780 (40,000 cells/well) were grown in a 96-well plate with a clear bottom (Greiner Bio-One, Frickenhausen, Germany) and incubated overnight. To assess internalization, the cells were treated with SNAP-Surface Alexa Fluor 647-labeled scFv-SNAP fusion proteins (1 μg in 200 μL of PBS) at 37 °C. For binding visualization, the cells were incubated with the same fusion proteins at 4 °C for 1 h. After two washing steps with PBS, the cells were stained with Hoechst 33,342 fluorescent nuclear counterstain (1:500 in PBS) (Thermo Fisher Scientific, Darmstadt, Germany) at room temperature for 15 min, followed by two washing steps. Fluorescence imaging was performed using a DMi8 S Live-cell microscope (Leica Microsystems, Wetzlar, Germany) using a 100 × oil immersion objective.

### 2.7 Cell Viability Assay of MMAE-Based ADCs

The cytotoxicity of MMAE-based ADCs was evaluated using a Cell Proliferation (XTT) Kit II (Roche, Mannheim, Germany) according to the manufacturer’s instructions. Ovarian cancer cells (5,000 cells/well) were seeded in a 96-well plate and incubated overnight at 37 °C. The cells were then treated with different concentrations of ADCs, naked scFv-SNAP fusion proteins, or free MMAE (0, 10, 20, 40, 80, 160, 320, 640 nM in 100 μL culture medium) for 72 h at 37 °C. Cells treated with PBS or zeocin were served as negative or toxic controls, respectively. The cytotoxicity was determined following manufacturer’s instructions by adding 50 μL of XTT labeling mixture to each well and incubating at 37 °C for 4 h. The substrate conversion was measured by monitoring the absorbance at 450 nm absorbance wavelength and 650 nm reference wavelength using an Infinite MPlex microplate reader (Tecan, Grödig, Austria). The experiment was carried out independently in triplicate and repeated three times.

### 2.8 Apoptosis Assay

The annexin V (AV)-SNAP fusion protein labeled with SNAP-Surface Alexa Fluor 647 (AV-647)^28^ was used to identify apoptotic cells, while propidium iodide (PI, Thermo Fisher Scientific) was used to detect necrotic cells. Ovarian cancer cells were seeded in 24-well plates at a density of 50,000 cells/well and incubated overnight. The cells were then treated with 640 nM MMAE-based ADCs, naked scFv-SNAP fusion proteins, or free MMAE at 37 °C for 48 h. Negative and positive controls were prepared by treating cells with PBS or camptothecin (Merck KGaA, Darmstadt, Germany), respectively. After harvesting, the cells were washed twice with AV binding buffer (10 mM HEPES, 140 mM NaCl, 2.5 mM CaCl_2_, pH 7.4) and stained with 0.5 μg of AV-647 at room temperature for 30 min in the dark. Following an additional wash, the cells were resuspended in 100 μL of AV binding buffer and stained with 0.1 μg of PI at room temperature for 10 min. Early and late apoptotic cells were analyzed by using a BD FACSCanto TM II Flow Cytometer. The experiment was repeated independently three times in triplicate.

### 2.9 Statistical Analysis

To validate and compare the toxicity of ADCs, the half maximal inhibitory concentration (IC_50_) was calculated by GraphPad Prism 9.0.0 (GraphPad Software, San Diego, CA, USA) and presented as the mean ± standard error of the mean (SEM). The proportions of apoptotic cells induced by different drugs were also shown as mean ± SEM. Statistical analysis was carried out using one-way ANOVA and corrected by Tukey’s test using GraphPad Prism 9.0.0. A *p*-value of *p* < 0.05 was considered statistically significant.

## 3 Results

### 3.1 Generation of EGFR-, Her2-, Trop2-, and TF-Specific ADCs

The scFv-SNAP fusion proteins were successfully produced by using a mammalian expression system in HEK293T cells. The proteins secreted in the cell culture medium were purified and enriched through His-tagged protein purification, utilizing the interaction between the His-tag and Ni-NTA, yielding 8–10 mg of fusion protein per liter of culture supernatant. As an example, the presence of scFv-Erbitux-SNAP in the cell culture medium was confirmed by SNAP-Surface Alexa Fluor 488, while it was absent in the flow-through (Supporting Information Figure 2), indicating efficient binding to the Ni-NTA Superflow cartridge. Meanwhile, the nonspecific binding proteins were removed stepwise, and scFv-Erbitux-SNAP was mainly eluted using an elution buffer containing 250 mM imidazole. The loss during the purification and enrichment was negligible, as the signal of the sample eluted by 250 mM imidazole was much stronger than that with 40 mM imidazole, despite using only one-third of the sample volume compared to other lanes (Supporting Information Figure 2).

The SNAP-tag enabled the conjugation of scFvs to BG-modified substrates. In this study, the SNAP-tag-fused proteins were conjugated with SNAP-Surface Alexa Fluor 647 or BG-MMAE based on the site-specific labeling property of SNAP-tag technology (Figure 1a). The efficient conjugation of all four proteins was achieved under physiological conditions within 2 h. While the unconjugated proteins had the full activity to bind SNAP-Surface Alexa Fluor 488 (lane 1 for each protein, Figure 1b), the binding site was completely blocked after incubation with SNAP-Surface Alexa Fluor 647 or BG-MMAE, as indicated by the absence of the BG-488 signal (lanes 2–3 for each protein, Figure 1b).

**Figure 1:**
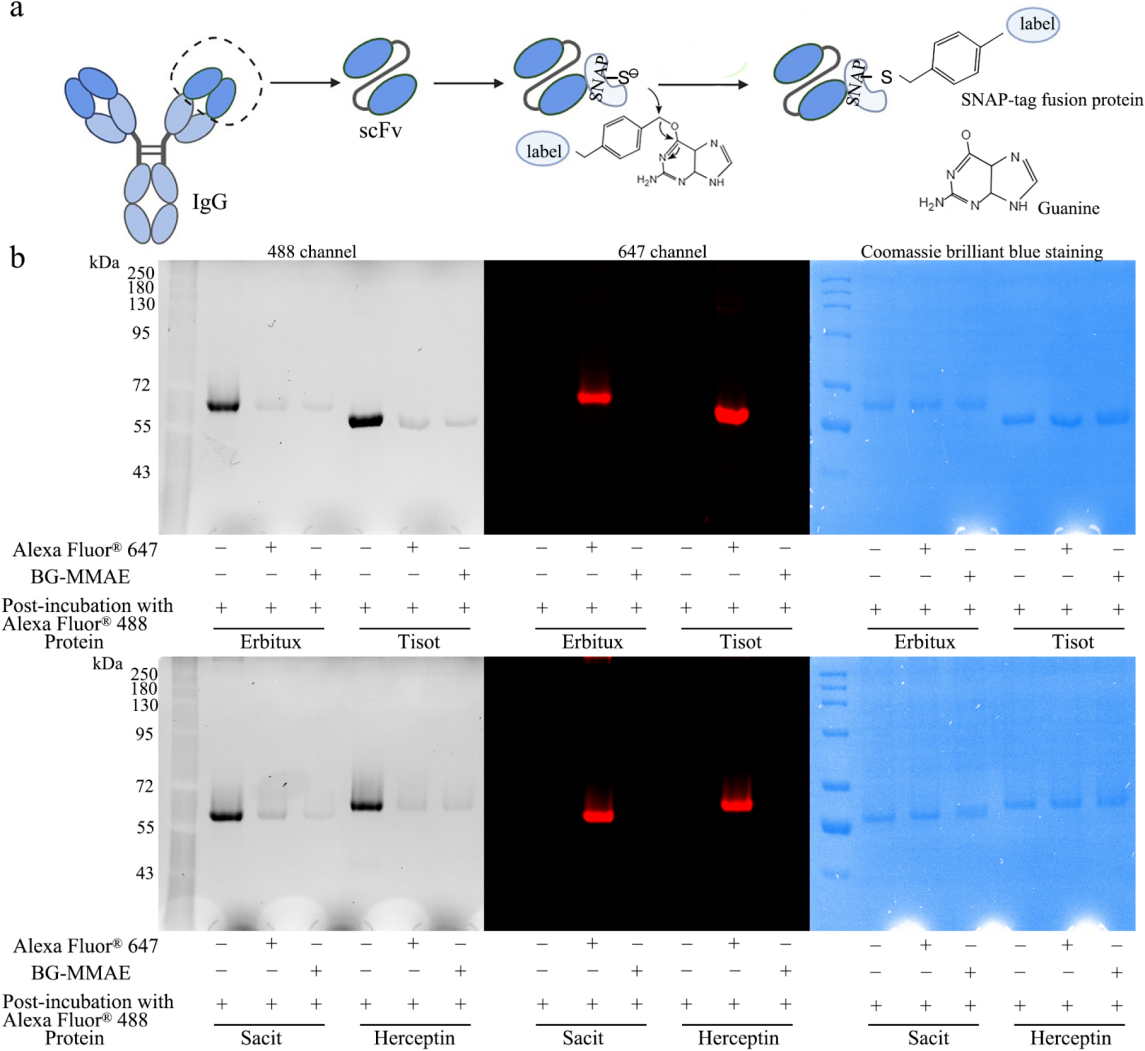
Efficient conjugation between SNAP-tag-fused proteins and BG-derivatives. (a) Schematic diagram illustrating scFv-SNAP-tag fusion proteins and their conjugation with BG-derivatives. (b) For each scFv-SNAP-tag fusion protein, three treatments were performed: (1) direct incubation with SNAP-Surface Alexa Fluor 488; (2) preincubation with SNAP-Surface Alexa Fluor 647 followed by incubation with SNAP-Surface Alexa Fluor 488; and (3) preincubation with BG-MMAE followed by incubation with SNAP-Surface Alexa Fluor 488. The fluorescence signals were visualized, and the corresponding Coomassie blue staining is shown.

### 3.2 Different Expression of EGFR, Her2, Trop2, and TF on the Surface of Ovarian Cancer Cells

Four ovarian cancer cell lines (SKOV3, OVCAR3, A2780, and OVCAR4) were used to evaluate the targeting properties of SNAP-tag-fused proteins against EGFR, Her2, Trop2, and TF. These four tumor-associated antigens were confirmed to be differently expressed on the cell surface (Figure 2). Among the cell lines, A2780 displayed minimal levels of all antigens. Both OVCAR4 and OVCAR3 expressed all four antigens, while SKOV3 expressed three antigens (EGFR, TF, and Her2) but lacked Trop2 expression. The details of the expression levels are listed in Table 1.

**Figure 2:**
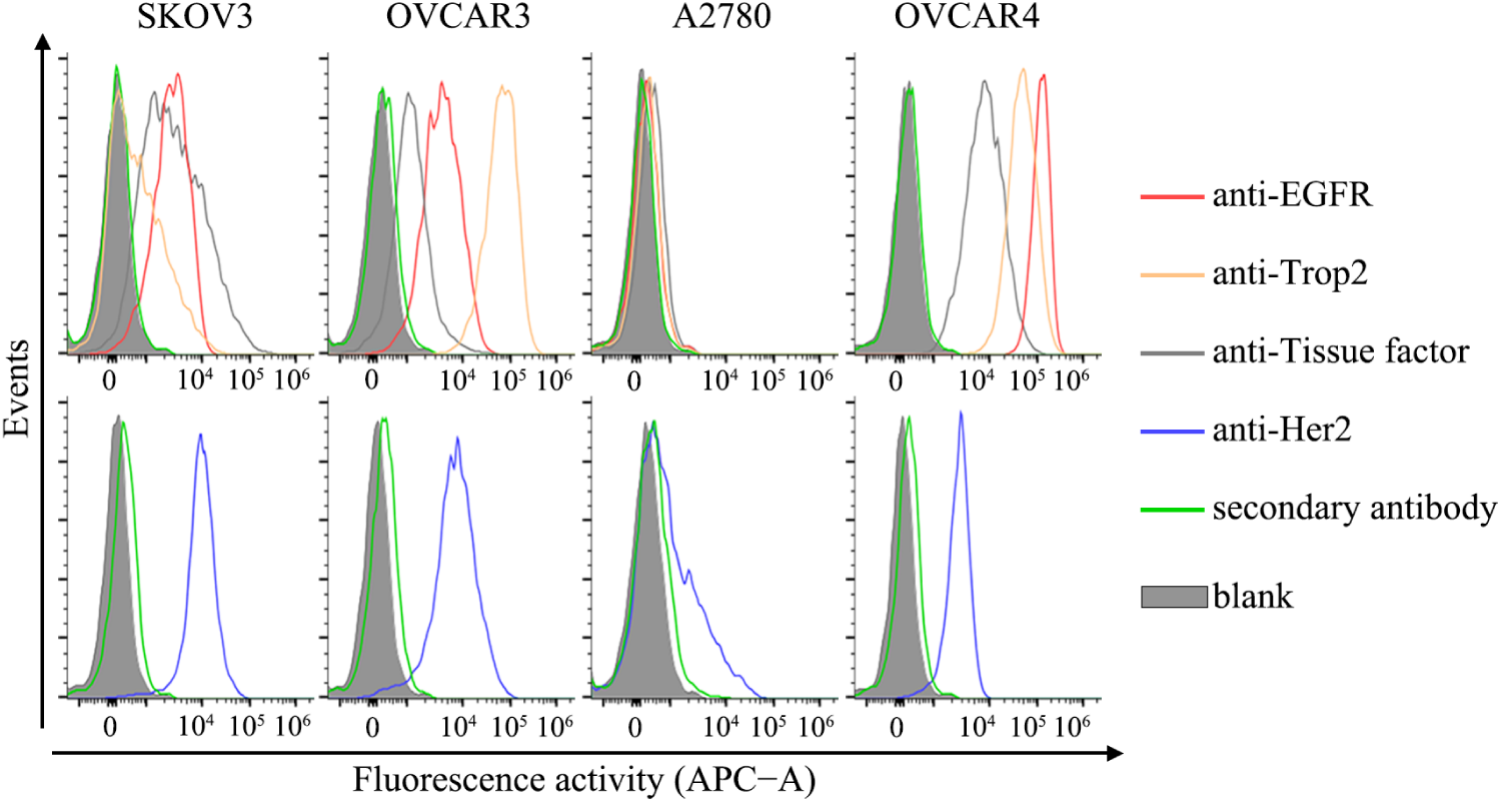
Different expression in ovarian cancer cell lines. The expression levels of EGFR, Trop2, TF, and Her2 in ovarian cancer cell lines were determined by flow cytometry. The cells were treated with anti-EGFR (EGFR monoclonal antibody, H11), anti-Trop2 (Trop2 monoclonal antibody, MR54), anti-TF (CD142 antibody), and anti-Her2 (ErbB2 monoclonal antibody, 3B5) antibodies, respectively, followed by incubation with a secondary antibody (Goat anti-Mouse IgG Highly Cross-Adsorbed Secondary Antibody, Alexa Fluor Plus 647). The cells were permeabilized to determine the Her2 expression according to manufacturer’s instructions.

**Table 1:** Expression Level for Trop-2, Her2, EGFR, and TF in Ovarian Cancer Cell Lines

	SKOV3	OVCAR3	A2780	OVCAR4
Trop-2	Low	High	Low	High
Her2	High	High	Low	Median
EGFR	Median	Median	Low	High
TF	Median	Median	Low	High

### 3.3 Binding of EGFR-, Her2-, Trop2-, and TF-Targeting scFv-SNAP-Tag Fusion Proteins to Antigen-Expressing Cells

The binding properties of SNAP-tag-fused scFv were evaluated on ovarian cancer cells expressing different levels of EGFR, Her2, Trop2, and TF. To better visualize the binding activity of the scFv-based ADCs, the scFvs were conjugated with SNAP-Surface Alexa Fluor 647 instead of BG-MMAE and validated by both flow cytometry and fluorescence microscopy. Consistent with the antigen expression profiles confirmed by commercial antibodies using flow cytometry, the scFv-SNAP-tag fusion proteins specifically bound to cells expressing EGFR (SKOV3, OVCAR3, and OVCAR4), Her2 (SKOV3, OVCAR3, and OVCAR4), Trop2 (OVCAR3 and OVCAR4), or TF (SKOV3, OVCAR3, and OVCAR4). Minimal binding was observed to cells with low expression of EGFR-, Her2-, Trop2-, and TF-low (A2780) or Trop2-low (SKOV3) cell lines (Figure 3a). Fluorescence microscopy further confirmed the binding process, showing that the scFvs quickly accumulated (within 1 h) and bound to the cell surface at 4 °C without nonspecific binding (Figure 3b).

**Figure 3:**
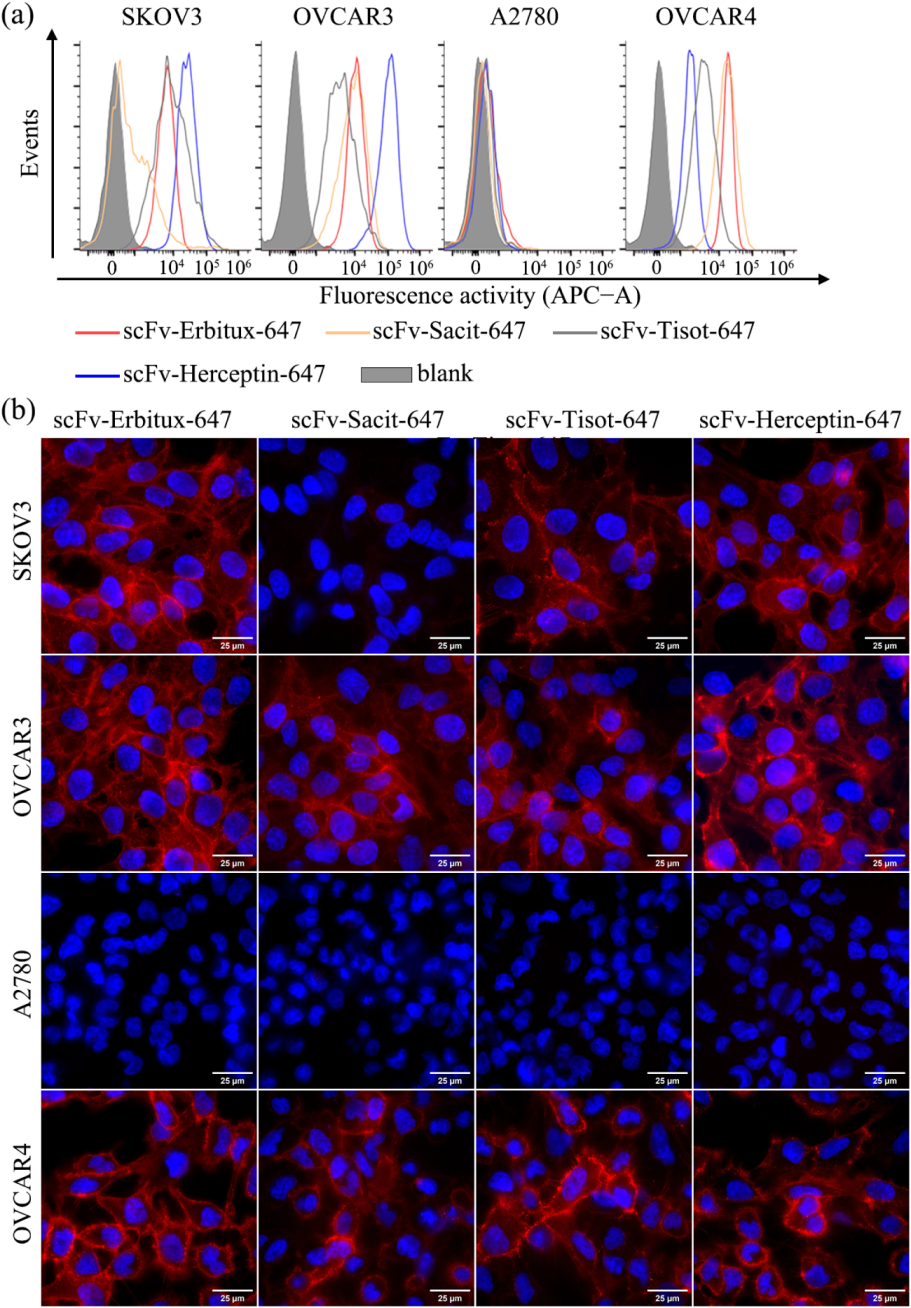
Specific binding of scFv-SNAP-tag fusion proteins to EGFR-, Trop2-, TF-, or Her2-expressing ovarian cancer cells. The specific binding properties of scFv-SNAP-tag fusion proteins were confirmed by (a) flow cytometry and (b) fluorescence microscopy. In (b), the red signal represents SNAP-Surface Alexa Fluor 647, while the blue signal represents Hoechst 33,342 nuclear counterstaining. The experiment was conducted at 4 °C.

### 3.4 Specific Internalization of EGFR-, Her2-, Trop2-, and TF-Targeting scFv-SNAP-Tag Fusion Proteins into Antigen-Expressing Cells

The internalization and the release of effectors within the cell are canonical mechanisms through which ADCs exhibit cytotoxicity. Therefore, determining the internalization property of an ADC is essential for its evaluation. In this study, cells were cultured and incubated with scFv-SNAP-tag fusion proteins at 37 °C to maintain cell activity. The internalization was visualized by fluorescence microscopy after 3 h incubation. Figure 4 shows that all scFv-SNAP-tag fusion proteins were specifically internalized into receptor-positive cell lines within 3 h, as indicated by white arrows. Some antibodies were still bound to the cell membrane. The rate of internalization varied between cell lines and antibodies. For instance, the scFv-Herceptin-647 internalized more slowly compared to the others, with less signal observed in the cytoplasm after 3 h. Fluorescence microscopy confirmed the rapid and specific internalization of scFv-SNAP-tag fusion proteins, with no nonspecific internalization observed in receptor-low cells.

**Figure 4:**
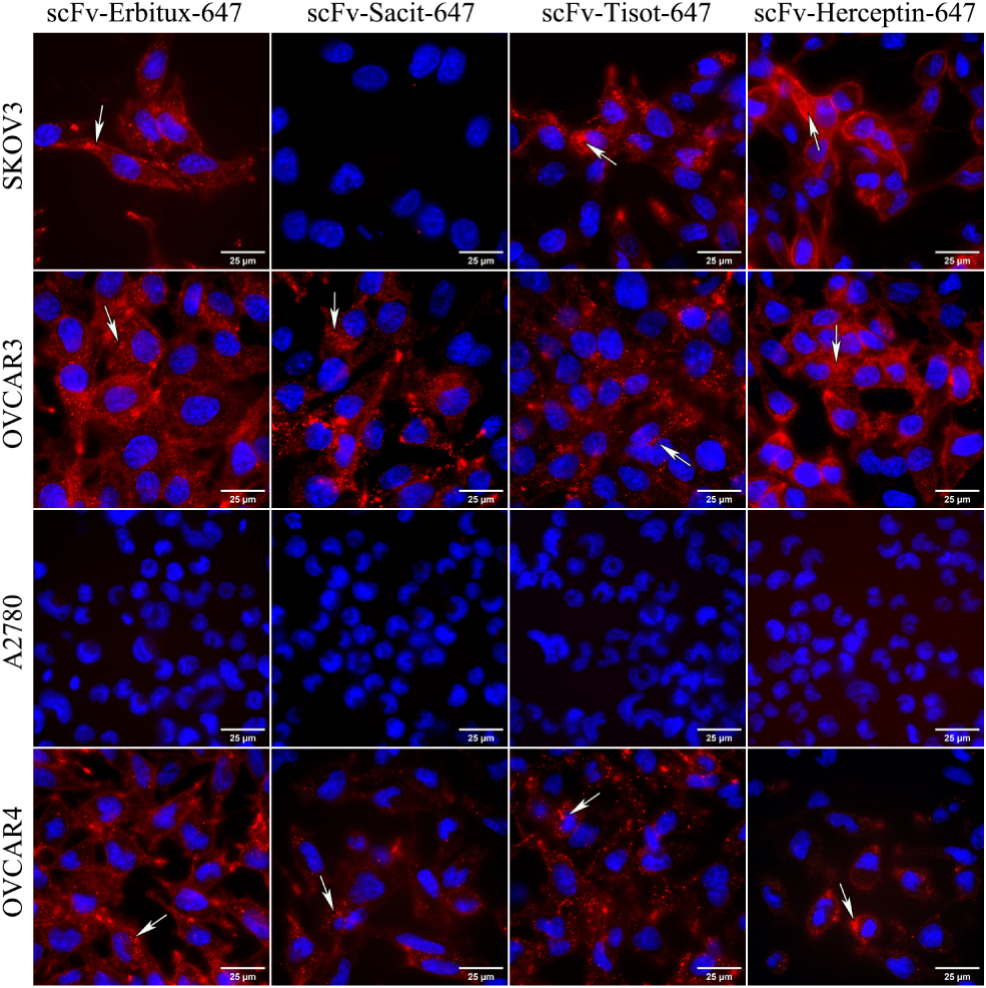
Specific and rapid internalization of the scFv-SNAP-tag fusion proteins. The cells were incubated with scFv-Erbitux-647, scFv-Sacit-647, scFv-Tisot-647, or scFv-Herceptin-647 at 37 °C for 3 h. The internalization was visualized by a fluorescence microscope. The red signal represents SNAP-Surface Alexa Fluor 647, while the blue signal represents Hoechst 33,342 nuclear counterstaining.

### 3.5 EGFR-, Her2-, Trop2-, and TF-Targeting SNAP-Tag-Based ADCs Exhibited Specific Cytotoxicity

A typical ADC should show cytotoxicity through the action of its payload following internalization. Evaluating an ADC commonly involves conjugating it with fluorescent dyes to visualize binding and internalization, along with using cell viability assays to measure dose-dependent cytotoxicity. To assess the cytotoxicity of the SNAP-tag-based ADCs, ovarian cancer cells were incubated with increasing concentrations of MMAE-based ADCs (0, 10, 20, 40, 80, 160, 320, 640 nM), and cell viability was measured after treatment (Figure 5).

**Figure 5:**
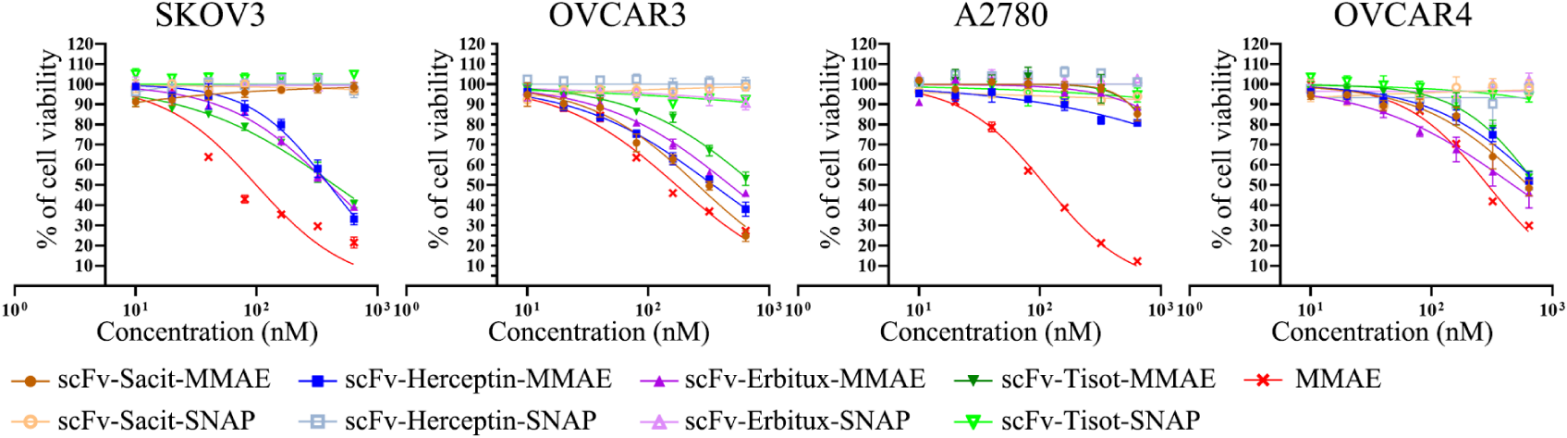
ScFv-MMAE induces specific cytotoxicity. The cells were incubated with increasing concentrations (0, 10, 20, 40, 80, 160, 320, 640 nM) of MMAE, scFv-MMAE, or scFv at 37 °C for 72 h. The cell viability was measured at each concentration. The experiment was repeated three times in triplicates, and the results are presented as mean ± SD of one independent measurement.

The cytotoxicity was observed in all cell lines after treatment with free MMAE due to its universal cell-killing property. Among the cell lines, SKOV3 was the most sensitive to MMAE (IC_50_: 96.6 ± 8.85 nM), while OVCAR4 was the least sensitive (IC_50_: 265 ± 18.95 nM). The scFv endowed MMAE with specificity, as the cytotoxicity was minimal in receptor-low cells. For example, the cell viability decreased with increasing concentration of scFv-Sacit-MMAE in OVCAR3 and OVCAR4 but remained unaffected in SKOV3 and A2780. Additionally, the scFv-SNAP-tag fusion proteins did not show any cytotoxicity even at the highest concentration (640 nM), indicating that the observed toxicity was entirely mediated by MMAE. The full details of the IC_50_ values are listed in Table 2.

**Table 2:** IC_50_ Values (nM) of Ovarian Cancer Cells Treated with scFv-MMAE, MMAE, and scFv^a^

	SKOV3	OVCAR3	A2780	OVCAR4
scFv-Sacit-MMAE	-	271 ± 13.93	-	538.5 ± 41.76
scFv-Sacit-SNAP	-	-	-	-
scFv-Herceptin-MMAE	407 ± 31.03	387.9 ± 21.63	-	870.1 ± 49.86
scFv-Herceptin-SNAP	-	-	-	-
scFv-Erbitux-MMAE	409.3 ± 36.87	415.4 ± 28.3	-	434.9 ±47.91
scFv-Erbitux-SNAP	-	-	-	-
scFv-Tisot-MMAE	499.5 ± 40.23	769.6 ± 48.44	-	739.6 ± 39.78
scFv-Tisot-SNAP	-	-	-	-
MMAE	96.6 ± 8.85	166 ± 12.26	127.2 ± 8.226	265 ± 18.95

a The experiment was repeated at least three times in triplicates. The data are presented as mean ± standard error of the mean (SEM).

### 3.6 The Cytotoxicity of EGFR-, Her2-, Trop2-, and TF-Targeting ADCs Was Induced by Apoptosis

Apoptosis is a form of programmed cell death. Previous studies have proved that MMAE can induce apoptosis, and MMAE-based ADCs also trigger cell death via apoptosis.^29,30^ In this study, we evaluated the proportion of apoptotic cells following treatment with EGFR-, Her2-, Trop2-, and TF-targeting ADCs. Figure 6a shows a scatter plot representing one measurement of Trop2-targeting ADC as an example. The four quadrants represent necrotic (Q1), late apoptotic (Q2), early apoptotic (Q3), and living (Q4) cells. For statistical analysis, the total apoptotic cell population was defined as the sum of the numbers of Q2 and Q3 (Figure 6b). In all cell lines, apoptosis was successfully induced by the positive control, camptothecin, indicating that none of the cell lines were exhibiting defects in the apoptotic pathway. Consistent with the cell viability assay results, MMAE alone induced nonspecific apoptosis. However, after conjugation with scFv-SNAP-tag fusion proteins, apoptosis was specifically induced in EGFR- (SKOV3, OVCAR3, and OVCAR4), Her2- (SKOV3, OVCAR3, and OVCAR4), Trop2- (OVCAR3 and OVCAR4), and TF-expressing cells (SKOV3, OVCAR3, and OVCAR4) (Figure 6a, Supporting Information Figures 3–5).

**Figure 6:**
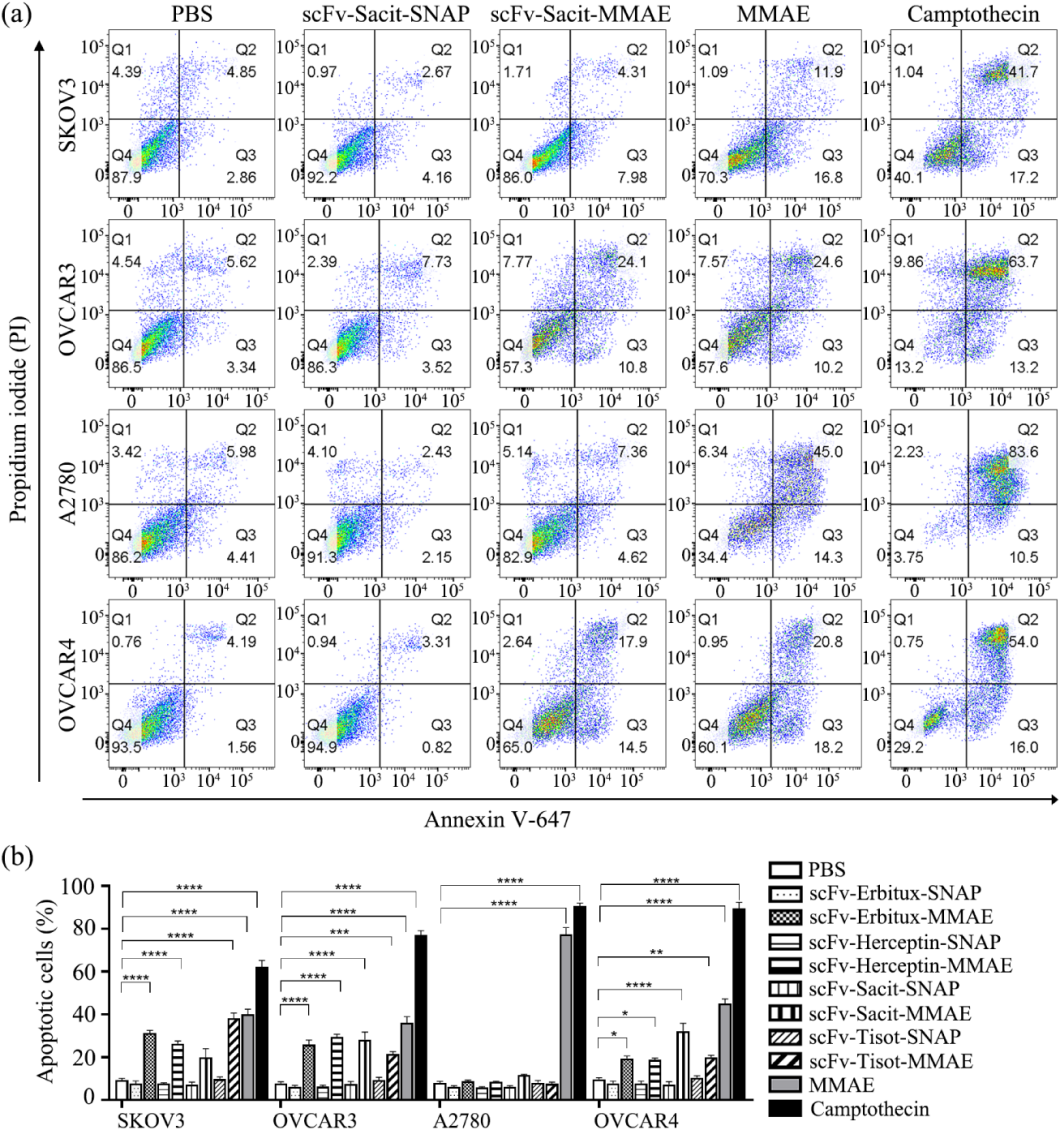
ScFv-SNAP-tag-MMAE induced apoptosis. (a) Scatter plot showing a representative measurement of Trop2-targeting ADC-inducing apoptosis. (b) Statistic analysis of apoptotic cells, including early (Q3) and late (Q2) apoptotic populations, following treatment with EGFR-, Her2-, Trop2-, and TF-targeting ADCs. The experiment was performed independently in triplicates, three times. Data are presented as mean ± SEM. The statistical analysis was conducted using one-way ANOVA and corrected by Tukey’s test (*p < 0.05, **** *p* < 0.0001).

## 4 Discussion

Ovarian cancer is a highly lethal malignant tumor, as most of the patients are diagnosed at an advanced stage due to the lack of effective early detection tools. After surgical resection, nearly all patients undergo paclitaxel plus platinum chemotherapy. Although the majority of patients initially responded well to platinum-based chemotherapy, over 70% experience recurrent relapses within 3 years due to acquired drug resistance.^31^ To improve the prognosis of ovarian cancer, the FDA approved targeted therapy—olaparib alone or in combination with bevacizumab—as first-line maintenance treatment of BRCA-mutated or homologous recombination-deficient-positive advanced ovarian cancer. However, olaparib is effective primarily in platinum-sensitive ovarian cancer, leaving platinum-resistant patients with limited treatment options. Mirvetuximab soravtansine, the first approved ADC for ovarian cancer, is applied for FRα-positive, platinum-resistant epithelial ovarian, fallopian tube, or primary peritoneal cancer. ADCs offer new hope for ovarian cancer patients and are also transforming the therapeutic landscape for many other cancer types.

Although FRα is a well-established target for ovarian cancer, its expression is heterogeneous across different tumor types and cell lines. Literature evidence indicates that EGFR, Her2, Trop2, and TF are expressed at moderate to high levels in commonly used ovarian cancer cell lines, such as SKOV3 and OVCAR3. Expanding ADC development to these alternative targets may therefore increase the applicability of targeted therapy and provide options for patients whose tumors express low levels of FRα.

To expand the application of ADCs in ovarian cancer treatment, we explored additional potential targets and generated scFv-Erbitux-SNAP, scFv-Herceptin-SNAP, scFv-Sacit-SNAP, and scFv-Tisot-SNAP since EGFR, Her2, Trop-2, and TF are also highly expressed in ovarian cancer. It is worthwhile to mention that the intact antibody was replaced with scFv in these constructs. As mentioned earlier, ADCs consist of three main components: a mAb, a linker, and a cytotoxic payload. The IgG architecture is the common backbone for ADCs due to its high affinity and specificity for tumor-associated antigens. However, the large size of full-length antibodies can hinder rapid penetration through the blood vessels. Additionally, the presence of two active binding sites increases the likelihood of the full-length antibody being captured by tumor cell surfaces .^32^ In contrast, scFvs are smaller and consist only of the variable regions of the heavy and light chains, which may allow for deeper tumor penetration due to their reduced size and lower affinity. The molecular weight cutoff for glomerular filtration is 30–50 kDa.^33^ Proteins ranging from 37 to 74 kDa are considered optimal for systemic delivery due to their longer half-lives in blood.^34^ By fusing the SNAP-tag to the scFv, the molecular weight of the scFv-based ADC increased to approximately 52 kDa, reducing the likelihood of rapid renal clearance and improving its half-life. Despite a decrease in affinity, the specificity of the scFv-based ADCs remains intact. In the context of Her2 as a model target, scFv-Herceptin-SNAP is expected to retain the antigen specificity of the parental Herceptin antibody but with reduced avidity and somewhat lower cell-binding intensity due to its monovalent format.^35,36^ Similar trends are observed with other therapeutic antibodies: Erbitux maintains high receptor affinity and robust cell binding through its intact IgG structure, whereas fragment-based derivatives exhibit reduced avidity and lack Fc-mediated functions. Sacituzumab govitecan illustrates how modifications of the IgG scaffold can alter pharmacokinetics and biodistribution while introducing new therapeutic properties, with a reported mean half-life of 15.3 h and a clearance rate of 0.14 L/h. In general, scFv constructs like scFv-SNAP exhibit faster clearance and shorter serum half-life compared to full-length IgGs, but offer advantages in modularity, rapid tissue penetration, and the potential for targeted delivery or imaging.

Extending this to therapeutic applications, scFv-SNAP-MMAE constructs targeting Her2, EGFR1, Trop-2, and tissue factor are expected to retain the specificity of their parental antibodies but may show slightly reduced avidity and cytotoxic potency due to the monovalent scFv format.^36^ Compared to full-length IgG-based ADCs such as Kadcyla, these scFv-SNAP-ADCs are anticipated to have faster systemic clearance and shorter serum half-life due to their smaller size and lack of Fc-mediated recycling.^37^ While this may limit circulation time, the modular nature of scFv-based ADCs can enhance tumor penetration, reduce off-target exposure, and provide flexibility for conjugation of different cytotoxic payloads, highlighting both their potential advantages and limitations relative to those of conventional IgG-based ADCs in ovarian cancer therapy.

The choice of MMAE as the cytotoxic payload warrants careful consideration, given its known bystander effect, whereby released MMAE can diffuse through cell membranes and affect neighboring cells, potentially leading to off-target toxicity. However, this property may also be therapeutically advantageous. In heterogeneous tumors, such as ovarian cancer, where not all cells uniformly express the target antigen, the bystander effect enables cytotoxic activity to extend beyond antigen-positive cells to adjacent antigen-negative tumor cells. This feature can enhance the overall efficacy of ADCs. Furthermore, the antibody component of the ADC facilitates selective accumulation within tumor tissue, which helps to minimize exposure to healthy tissues and reduces the risk of systemic toxicity. Thus, despite its potential drawbacks, MMAE remains a preferred payload in the present study due to its ability to maximize therapeutic benefits in the context of tumor heterogeneity.

Flow cytometry and fluorescence microscopy demonstrated that scFv-Erbitux-SNAP, scFv-Herceptin-SNAP, scFv-Sacit-SNAP, and scFv-Tisot-SNAP specifically bound to EGFR, Her2, Trop2, and TF, respectively. Interestingly, the scFv-SNAP-tag fusion proteins showed slightly weaker binding to the aforementioned OVCAR3 cells (Figure 3) compared with the corresponding commercial antibodies (Figure 2). This difference may be due to the relatively lower antigen expression in OVCAR3 cells and the monovalent nature of the scFv fragments, which inherently generate weaker signals than bivalent full-length antibodies. In OVCAR4 cells, where antigen expression is higher, robust scFv-SNAP-tag fusion protein binding was readily observed. Notably, these observations do not affect the overall conclusions regarding antigen-dependent binding specificity and functional activity of the scFv-SNAP-tag fusion proteins. All scFv-SNAP-tag fusion proteins were internalized within 3 h, indicating their potential for targeted delivery of drugs. While the toxicity of a conventional ADC primarily arises from the payload and immune response triggered by the Fc region of the mAb, some researchers argue that these immune reactions mediated may be unnecessary and their effects may be unpredictable.^38,39^ For instance, an assessment from the European Medicines Agency in 2012 reported that Adcetris shows no complement-dependent cytotoxicity and limited antibody-dependent cell-mediated cytotoxicity *in vitro*.^13^ By using scFv instead of a full-length antibody, immune-related toxicity is reduced, particularly off-target toxicity caused by FcγR-dependent uptake of ADC aggregates.^40^

The linker in an ADC serves as a smart connector between the mAb and the payload. An ideal linker maintains ADC stability during circulation in the bloodstream and releases the payload specifically at the tumor site. Linkers can be classified into cleavable linkers and noncleavable linkers. Generally, payloads released from ADCs with noncleavable linkers retain an amino acid appendage and the linker itself.^41^ Compared to cleavable linkers, noncleavable linkers are considered to not only enhance ADC stability in blood circulation but also limit the bystander effect.^41^ The bystander effect occurs when free cytotoxins are released from ADCs or diffused out of the target cell, allowing them to kill surrounding cells regardless of whether they express the target antigen. As a result, ADCs with noncleavable linkers are better suited for treating homogeneous cancer.^42^ Given the heterogeneous nature of ovarian cancer, we generated ADCs with the cleavable linker valine-citrulline, which should theoretically increase their toxicity.

The payload of an ADC is activated either upon release from the ADC or through antibody degradation, serving as the primary component responsible for the ADC’s cytotoxicity effects. As of this writing, 16 ADCs have been approved worldwide. Among these, MMAE is the most commonly used cytotoxic agent. MMAE inhibits cell division by blocking tubulin polymerization. Due to its potent toxicity (IC_50_: 0.47–6.5 nM),^43^ MMAE cannot be used as a standalone drug. However, conjugating MMAE to mAb expands the therapeutic window of both the mAb and MMAE itself. This conjugation lowers the minimum effective dose of the mAb while increasing the maximum tolerated dose of MMAE, as the mAb facilitates targeted delivery to tumor sites while sparing normal or nontarget cells.

In this study, we confirmed the expanded therapeutic window of SNAP-tag-based ADCs through a cytotoxic assay. Unconjugated, the MMAE displayed cytotoxicity in all tested cell lines, with IC_50_ ranges from 96.6 to 265 nM. Among these, SKOV3 was the most sensitive cell line to MMAE, while OVCAR4 was the least. In contrast, SNAP-fused scFvs alone did not exhibit cytotoxicity at concentrations up to 640 nM. After conjugation, the ADCs showed specific toxicity in EGFR-, Her2-, Trop2-, and TF-expressing cell lines while showing minimal toxicity in cell lines with low expression of these targets. Notably, the IC_50_ values of scFv-MMAE (IC_50_: 271–870.1 nM) were significantly higher than those of free MMAE, indicating an increased maximum tolerated dose. Furthermore, the conjugation of MMAE to scFvs conferred cytotoxicity, reflecting a reduced minimum effective dose.

In addition to the three main components of an ADC, the conjugation methods also play a crucial role in ADC design. Conjugation methods have evolved through three generations. Compared to the first and second generation ADCs, which rely on random conjugation through lysines or reduced interchain cysteines, resulting in heterogeneous products, the third generation focuses on site-specific conjugation to produce homogeneous products with a defined drug-to-antibody ratio (DAR).^32^ Enzymatic methods for site-specific antibody conjugation have gained considerable attention due to their precision and mild reaction conditions, including sortase A-mediated transpeptidation, microbial transglutaminase-catalyzed modification, and glycosyltransferase-based strategies. While these approaches offer site selectivity, they also suffer from limitations such as low reaction efficiency, substrate restrictions, or the need for extensive protein engineering.^44^ In contrast, the SNAP-tag technology enables rapid and irreversible conjugation with benzylguanine-modified substrates in a stoichiometric and highly efficient manner. This technology allows the generation of homogeneous ADCs, reducing heterogeneity and simplifying downstream characterization. The robustness, versatility, and cost-effectiveness of the SNAP-tag make it a particularly attractive platform for developing next-generation ADCs. However, as a relatively large fusion tag (∼20 kDa), SNAP-tag may influence protein folding, pharmacokinetics, or immunogenicity.^45^ All of these issues, which are also disadvantages of other enzymatic methods, should be carefully addressed to enable future clinical translation or commercial development. In this study, we confirmed that SNAP-tag technology enables highly efficient conjugation within 2 h under physiological conditions with a 1:1 stoichiometry. This method avoids complex steps, such as the redox reaction, the use of additional catalytic materials, or the consumption of excess cytotoxic payload. With a single SNAP-tag, our ADC can deliver only one payload molecule. While increasing the number of payloads is expected to enhance cytotoxicity, more is not always better. A high DAR can lead to rapid clearance due to increased hydrophobicity.^46^

DAR 2–4 was generally considered optimal for ADC, and studies have shown that mAb with two MMAE molecules exhibit the best response when the dose is doubled to match the total amount of mAb with 4 or 8 molecules of MMAE *in vivo*.^47^ However, no universal standard for the ADC design exists. Given the structure of our scFv-SNAP, loading too many payloads may accelerate clearance and reduce potency. Therefore, we designed our scFv-SNAP with a single SNAP-tag rather than by incorporating two or more.

All in all, we generated four scFv-SNAP fusion proteins targeting EGFR, Her2, Trop2, and TF, and we conducted a series of *in vitro* assays to examine their antigen-dependent binding and internalization patterns and payload-mediated cytotoxicity. The results presented here demonstrated qualitative and target-dependent cellular recognition and functional activity. However, several important limitations remain and substantially restrict the extent of the conclusions that can be drawn from the current data set.

First, although SNAP-tag chemistry enables stoichiometric conjugation under mild conditions, our study did not include essential biochemical analyses such as size exclusion chromatography (SEC) or mass spectrometry. These experiments are required to confirm protein homogeneity, molecular integrity, and especially the oligomerization state of the scFv-SNAP constructs. Given that scFv fragments frequently form dimers or higher-order oligomers, the absence of SEC data precludes a definitive assessment of whether the constructs used here were monomeric. This uncertainty directly affects the interpretation of antigen binding, internalization, and cytotoxicity results, as multivalent species could introduce avidity effects that could not be attributed to a monovalent scFv format. Therefore, the present findings should be regarded as preliminary observations obtained from protein preparations whose biophysical properties remain incompletely characterized. Second, while flow cytometry and fluorescence microscopy in ovarian cancer cell lines provided qualitative evidence of antigen-dependent recognition, we did not perform quantitative measurements of the affinity or binding kinetics. Competition assays using purified antigens or biophysical analyses are required to rigorously evaluate specificity and affinity and to compare scFv-SNAP constructs with their parental antibodies. Without these data, it is not possible to conclude whether the binding properties of the scFv fusion proteins reflect their intrinsic monovalent characteristics, are influenced by potential oligomerization, or retain the affinity levels expected from the parental antibodies. Third, although the cytotoxicity assays demonstrated antigen-dependent activity of the scFv-MMAE constructs, these findings alone do not allow conclusions regarding improved tumor penetration, design optimization, or suitability for therapeutic development. Additionally, no *in vivo* pharmacokinetic, biodistribution, or efficacy studies were performed. Taken together, the findings presented here indicated only that scFv-SNAP fusion proteins can be produced, conjugated through SNAP-tag chemistry, and evaluated qualitatively in cellular assays. The absence of SEC, mass spectrometry, quantitative affinity measurements, and *in vivo* validation substantially limits the interpretability of the data and prevents a reliable assessment of the suitability of these constructs as ADC candidates. Future studies will require comprehensive biochemical characterization to determine monomeric purity and structural integrity, quantitative binding analyses to establish specificity and affinity, and *in vivo* experiments to evaluate the pharmacokinetics, tumor distribution, and therapeutic index. Thus, the constructs described here were designed as a proof-of-concept to establish the feasibility of scFv-SNAP-based ADCs, and there still remains a lot of work to be done in the future.

## Supplementary Material



## Notes

Amino acid sequence for scFv-SNAP molecules and additional figures (Figures S1–S5) (PDF)
