# Supplementary material for: SNAP-Tag-Based Antibody–Drug Conjugates Targeting Epidermal Growth Factor Receptor 1, Epidermal Growth Factor Receptor 2, Trophoblast Cell-Surface Antigen 2, and Tissue Factor for Ovarian Cancer Treatment
Source: ACS Omega. 2026 Mar 10;11(11):17393–403. doi: 10.1021/acsomega.5c10377 (PMC13019189; doi:10.1021/acsomega.5c10377)
Supplement: Supplementary file 1 [file ao5c10377_si_001.pdf]

SNAP-tag based antibody drug conjugates targeting epidermal growth factor receptor 1, epidermal growth factor receptor 2, trophoblast cell-surface antigen 2 and tissue factor for ovarian cancer treatment

Chaoyu Zhang[1], Wenjie Sheng[1], T. M. Mohiuddin[1,2], Marwah Al-Rawe[1], Roland Schmitz[3], Marcus Niebert[3], Felix Zeppernick[1], Ivo Meinhold-Heerlein[1], and Ahmad Fawzi Hussain[1]*

[1] Department of Gynecology and Obstetrics, Medical Faculty, Justus-Liebig-University Giessen, Klinikstr. 33, 35392 Giessen, Germany

[2] Brandenburg, Medizinische Hochschule Brandenburg Campus GmbH, Hochstraße 29, 14770 Brandenburg an der Havel

[3] Institute of Pathology, University Hospital Giessen, Justus-Liebig-University Giessen, Langhanssstr. 10, 35392 Giessen, Germany.

* Correspondence: ahmad.f.hussain@gyn.med.uni-giessen.de

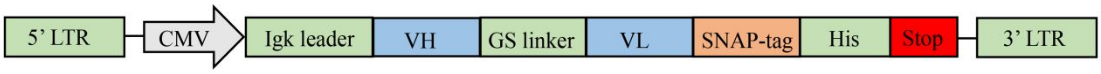

Supplementary Figure 1. Schematic of scFv-SNAP construct design. CMV: cytomegalovirus enhancer and promoter; IgK leader: murine immunoglobulin kappa chain leader; VH: variable heavy chain; GS linker: glycine-serine linker; VL: variable light chain; His: polyhistidin tag; Stop: TGA stop codon.

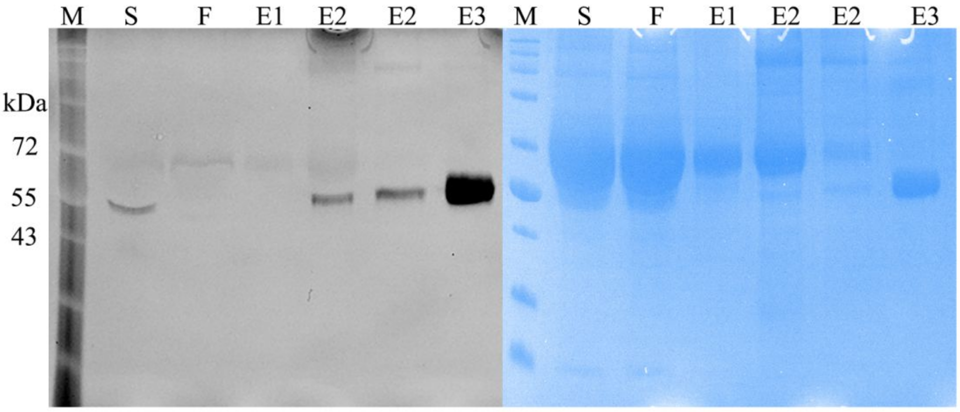

Supplementary Figure 2. Purification and enrichment of scFv-Erbiux-SNAP. The scFv-Erbitux-SNAP was purified and enriched from culture medium with increasing concentration of imidazole. Fractions were incubated with SNAP-Surface Alexa Fluor 488 (left) followed by Coomassie blue staining (right). M: Blue prestained protein standard broad range (11-250 kDa); S: Supernatant of culture medium; F: Flowthrough of supernatant; E1-E3: Flow-through of washing buffer containing different concentration of imidazole (10, 40 and 250 mM imidazole, respectively).

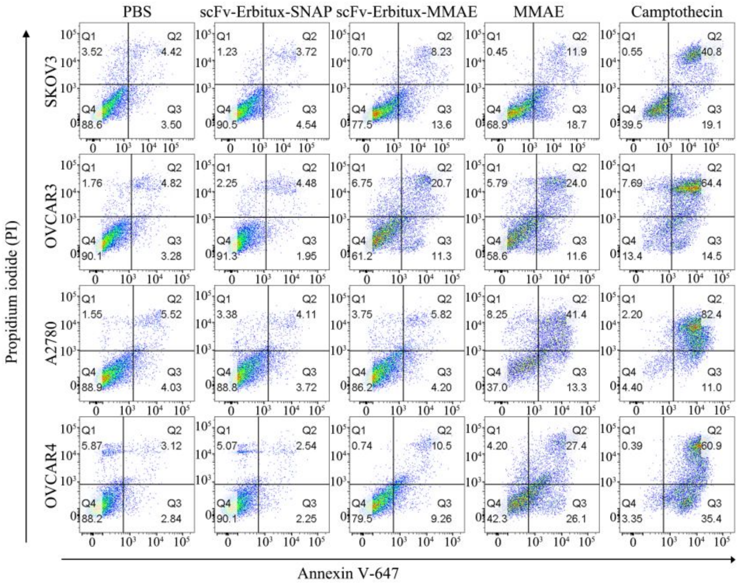

Supplementary Figure 3. Scatter plot showing a representative measurement of EGFR-targeting ADC-inducing apoptosis.

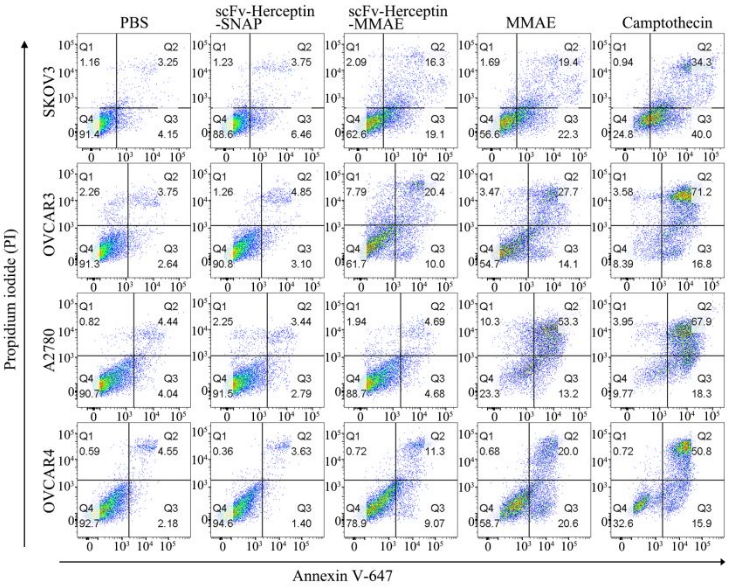

Supplementary Figure 4. Scatter plot showing a representative measurement of Her2-targeting ADC-inducing apoptosis.

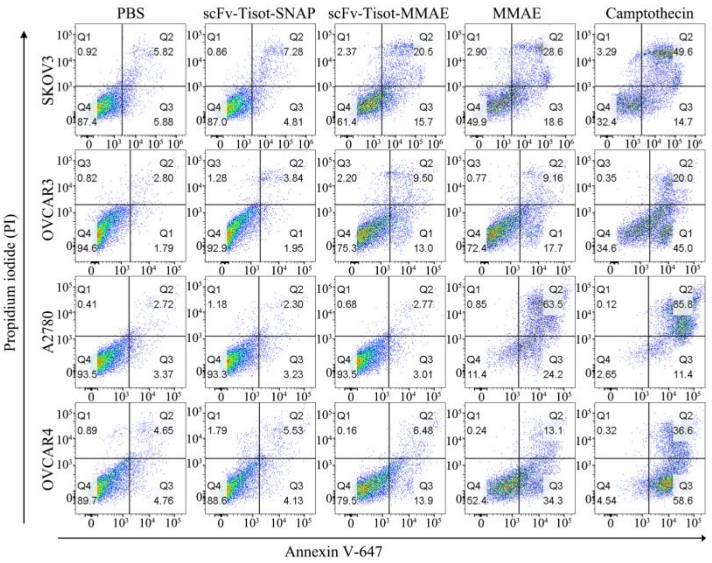

Supplementary Figure 5. Scatter plot showing a representative measurement of TF-targeting ADC-inducing apoptosis.

Amino acid sequence for scFv-SNAP molecules

scFv-Erbitux-SNAP

QVQLKQSGPGLVQPSQSLSITCTVSGFSLTNYGVHWVRQSPGKGLEWLGVIWSGGNTDY
NTPFTSRLSINKDNSKSQVFFKMNSLQSNDTAIYYCARALTYYDYEFAYWGQGTLVTVS
AGGGGSGGGGSGGGGSDILLTQSPVILSVSPGERVSFSCRASQSIGTNIHWYQQRTNGSPR
LLIKYASESISGIPSRFSGSGSGTDFTLSINSVESEDIADYYCQQNNNWPTTFGAGTKLELK
GSSRMDKDCEMKRTTLDSPLGKLELSGCEQGLHEIKLLGKGTSAADAVEVPAPAAVLGG
PEPLMQATAWLNAYFHQPEAIEEFPVPALHHPVFQQESFTRQVLWKLLKVVKFGEVISYQ

QLAALAGNPAATAAVKTALSGNPVPILIPCHRVVSSSGAVGGYEGGLAVKEWLLAHEGH
RLGKPGL

scFv-Herceptin-SNAP

EVQLVESGGGLVQPGGSLRLSCAASGFNIKDTYIHWVRQAPGKGLEWVARIYPTNGYTR
YADSVKGRFTISADTSKNTAYLQMNSLRAEDTAVYYCSRWGGDGFYAMDYWGQGTLV
TVSSGGGGSGGGGSGGGGSDIQMTQSPSSLSASVGDRVTITCRASQDVNTAVAWYQQKP
GKAPKLLIYSASFLYSGVPSRFSGSRSGTDFTLTISSLQPEDFATYYCQQHYTTPPTFGQGT
KVEIKSRMDKDCEMKRTTLDSPLGKLELSGCEQGLHEIKLLGKGTSAADAVEVPAPAAV
LGGPEPLMQATAWLNAYFHQPEAIEEFPVPALHHPVFQQESFTRQVLWKLLKVVKFGEVI
SYQQLAALAGNPAATAAVKTALSGNPVPILIPCHRVVSSSGAVGGYEGGLAVKEWLLAH
EGHRLGKPGL

scFv-Sacit-SNAP

QVQLQQSGSELKKPGASVKVSCKASGYTFTNYGMNWVKQAPGQGLKWMGWINTYTGE
PTYTDDFKGRFAFSLDTSVSTAYLQISSLKADDTAVYFCARGGFGSSYWYFDVWGQGSL
VTVSSGGGGSGGGGSGGGGSDIQLTQSPSSLSASVGDRVSITCKASQDVSIAVAWYQQKP
GKAPKLLIYSASYRYTGVPDRFSGSGSGTDFTLTISSLQPEDFAVYYCQQHYITPLTFGAGT
KVEIKSRMDKDCEMKRTTLDSPLGKLELSGCEQGLHEIKLLGKGTSAADAVEVPAPAAV
LGGPEPLMQATAWLNAYFHQPEAIEEFPVPALHHPVFQQESFTRQVLWKLLKVVKFGEVI
SYQQLAALAGNPAATAAVKTALSGNPVPILIPCHRVVSSSGAVGGYEGGLAVKEWLLAH
EGHRLGKPGL

scFv-Tisot-SNAP
EVQLLESGGGLVQPGGSLRLSCAASGFTFSNYAMSWVRQAPGKGLEWVSSISGSGDYTY
YTDSVKGRFTISRDNSKNTLYLQMNSLRAEDTAVYYCARSPWGYYLDSWGQGTLVTVS
SGGGGSGGGGSGGGGSDIQMTQSPPSLSASAGDRVTITCRASQGISSRLAWYQQKPEKAP
KSLIYAASSLQSGVPSRFSGSGSGTDFTLTISSLQPEDFATYYCQQYNSYPYTFGQGTKLEI
KSRMDKDCEMKRTTLDSPLGKLELSGCEQGLHEIKLLGKGTSAADAVEVPAPAAVLGGP
EPLMQATAWLNAYFHQPEAIEEFPVPALHHPVFQQESFTRQVLWKLLKVVKFGEVISYQ
QLAALAGNPAATAAVKTALSGNPVPILIPCHRVVSSSGAVGGYEGGLAVKEWLLAHEGH
RLGKPGL
